# On the Geographical Distribution of Tropical Diseases in Africa

**Published:** 1895-09

**Authors:** 


*On the Geographical Distribution of Tropical Diseases in Africa.* By R. W. FELKIN, M.D. Pp. 79. Edinburgh: WILLIAM F. CLAY. 1895.

Dr. Felkin divides the vast area of Africa into eight divisions, and adopts a system of symbols which by their shape, colour and number indicate the presence, the prevalence or the intense prevalence of the diseases common to each region. The reader has therefore only to master the significance of the symbols, and then by looking at the important map which is given he can see at a glance the insalubrity or otherwise of any particular region.

It is needless to say that the pink colour indicating malarial districts is by far the most prevalent, especially on the West, South-West, and East Coasts, and on that immense belt of low-lying water-logged land which forms the barrier between the sea and the high plateau in the Interior and which has been truly named the Grave of the White Man.

There are short descriptions given in the work of the various diseases in each region with some remarks on treatment.

The author is a firm believer in the microbe discovered by Laveran as the cause of malarial fever. He considers that Egyptian Chlorosis and Bèri-Bèri, to which it is akin, are caused by the larvæ of anchylostomum duodenale. He does not consider yellow fever to be a malarial disease at all, and that it requires for its development only a certain temperature and a certain amount of moisture in the air—a temperature of 68° to 70° F. and 74 per cent. of moisture. He regards it as a disease of filth, which rarely leaves the sea coasts and the mouths of rivers.

Dr. Felkin strongly advocates the use of large doses of ipecacuanha in the treatment of tropical dysentery; but we regret he did not acknowledge the merit (one which has met with scanty reward), due to Edward Scott Docker, late Surgeon of H.M. 18th Hussars, the originator of that treatment, by which the mortality of the disease has been greatly reduced.

Upon the whole this is a useful little book.

